# Monstrosities in General: A Double-Faced Horse

**Published:** 1882-07

**Authors:** 


MONSTROSITIES IN GENERAL: A DOUBLE-FACED HORSE.—A very rare deformity, classed technically as *tetrascelus bifacialis conjunctus*, is described by Dr. Th. Bauer (*Zeitschrift f. Thiermedicin*) as occurring in a foal. The mother was seven years old, and the birth was normal.

The top of the skull was double, there were four eyes, two ears, one lower jaw, slightly twisted outwards, one set of cervical vertebræ. At the first dorsal vertebræ the backbone split so that two spines were formed. There was therefore a double chest, with two sets of ribs. The animal had two pelves united together, but only four feet and only one anus. There were two tongues and two throats, but only one larnyx and two nasal openings. Only one gullet and trachea were present.

There have been only ten such cases as the above reported in domestic animals, viz., six in sheep, two in pigs, one in a cat, and one in a horse. Also two in fowls.

The cause of such monstrosities is thought to be in a crossing or partial fusing of two primitive traces. Dr. E. P. Murdock, in a recent lecture on this subject, makes some comments which may be appropriately quoted here: "I have often observed," he says, "that peculiar kinds of deformities are peculiar to certain orders of the animal kingdom, such as supernumerary prongs on the echinodermata, cleft caudal appendages in the fish, extra limbs or double feet in birds, acephalous monsters in sheep, cyclops in swine, and double monsters in man. It is also true that monstrosities are much more frequent in domestic animals than in the wild state. This is also true of plants spoiled by cultivation. They are more frequent in mixed races than in isolated tribes, and more frequent in the female than in the male sex. Even in birds they are more frequent in the pure family classes.

